# Associations between self‐reported sleep quality and white matter in community‐dwelling older adults: A prospective cohort study

**DOI:** 10.1002/hbm.23739

**Published:** 2017-07-26

**Authors:** Claire E. Sexton, Enikő Zsoldos, Nicola Filippini, Ludovica Griffanti, Anderson Winkler, Abda Mahmood, Charlotte L. Allan, Anya Topiwala, Simon D. Kyle, Kai Spiegelhalder, Archana Singh‐Manoux, Mika Kivimaki, Clare E. Mackay, Heidi Johansen‐Berg, Klaus P. Ebmeier

**Affiliations:** ^1^ FMRIB Centre Nuffield Department of Clinical Neurosciences, University of Oxford Oxford United Kingdom; ^2^ Department of Psychiatry University of Oxford Oxford United Kingdom; ^3^ Sleep and Circadian Neuroscience Institute Nuffield Department of Clinical Neurosciences, University of Oxford Oxford United Kingdom; ^4^ Department of Psychiatry and Psychotherapy Medical Center ‐ University of Freiburg, Faculty of Medicine, University of Freiburg Germany; ^5^ INSERM, U1018, Centre for Research in Epidemiology and Population Health France; ^6^ Department of Epidemiology and Public Health University College London London United Kingdom

**Keywords:** brain, cognition, diffusion tensor imaging, executive function, insomnia, magnetic resonance imaging, memory, processing speed

## Abstract

Both sleep disturbances and decline in white matter microstructure are commonly observed in ageing populations, as well as in age‐related psychiatric and neurological illnesses. A relationship between sleep and white matter microstructure may underlie such relationships, but few imaging studies have directly examined this hypothesis. In a study of 448 community‐dwelling members of the Whitehall II Imaging Sub‐Study aged between 60 and 82 years (90 female, mean age 69.2 ± 5.1 years), we used the magnetic resonance imaging technique diffusion tensor imaging to examine the relationship between self‐reported sleep quality and white matter microstructure. Poor sleep quality at the time of the diffusion tensor imaging scan was associated with reduced global fractional anisotropy and increased global axial diffusivity and radial diffusivity values, with small effect sizes. Voxel‐wise analysis showed that widespread frontal‐subcortical tracts, encompassing regions previously reported as altered in insomnia, were affected. Radial diffusivity findings remained significant after additional correction for demographics, general cognition, health, and lifestyle measures. No significant differences in general cognitive function, executive function, memory, or processing speed were detected between good and poor sleep quality groups. The number of times participants reported poor sleep quality over five time‐points spanning a 16‐year period was not associated with white matter measures. In conclusion, these data demonstrate that current sleep quality is linked to white matter microstructure. Small effect sizes may limit the extent to which poor sleep is a promising modifiable factor that may maintain, or even improve, white matter microstructure in ageing. *Hum Brain Mapp 38:5465–5473, 2017*. © **2017 Wiley Periodicals, Inc.**

## INTRODUCTION

There is growing evidence to support a role for sleep in the maintenance of the brain's white matter. Gene expression studies have shown that brain transcripts involved in myelin synthesis or maintenance are up‐regulated during sleep [Bellesi, 2015], with the proliferation of oligodendrocyte precursor cells doubling during sleep [Bellesi et al., 2013]. Diffusion tensor imaging (DTI) studies have documented reduced fractional anisotropy (FA) and increased radial diffusivity (RD) within fronto‐subcortical tracts in insomnia [Li et al., 2016; Spiegelhalder et al., 2014]. Although the relationship between DTI metrics is complex, a signature of reduced FA and increased RD can reflect microstructural changes associated with white matter degeneration, such as reduced axon density or reduced myelin [Zatorre et al., 2012].

Given that sleep disturbances are reported by approximately 50% of older adults [Neikrug and Ancoli‐Israel, 2010], poor sleep could be a promising target in the search for modifiable factors that could help maintain, or improve, white matter microstructure in ageing. Indeed, poor sleep quality has been associated with the presence and severity of white matter hyperintensities (WMH) [Del Brutto et al., 2015], and higher sleep fragmentation associated with reduced FA and higher RD values [Baillet et al., 2017]. However, it is unclear if there is a continuum of white matter changes with increasing persistence of symptoms.

We use DTI to examine the relationship between sleep quality and white matter microstructure in a UK community‐dwelling cohort [Filippini et al., 2014]. We hypothesize that both current poor sleep quality and persistent poor sleep quality over a 16‐year period will be associated with a pattern of reduced FA and increased RD in fronto‐subcortical regions.

## METHODS

### Participants

The sample was drawn from participants recruited to take part in the Whitehall II Imaging Sub‐Study between May 2012 and December 2014 [Filippini et al., 2014]. All participants were members of the Whitehall II study, a prospective occupational cohort study established in 1985 [Marmot and Brunner, 2005]. Ethical approval was obtained from the University of Oxford Central University Research Ethics Committee, and the UCL Medical School Committee on the Ethics of Human Research. Informed written consent was obtained from all participants.

Eligible participants reported no history of dementia or neurological illness, did not display significant abnormalities on structural MRI scans, did not report current sleep apnoea or prescribed medication for a sleep disorder, and had complete data on sleep and DTI measures.

### Assessment of Sleep Quality

Sleep quality was assessed at the same time‐point as the MRI scan using the Pittsburgh Sleep Quality Index (PSQI) [Buysse et al., 1989], a self‐rated questionnaire made up of seven component scores that assess sleep quality and disturbance over the previous month. A threshold of 6 was used to classify participants as good sleepers (< 6) or poor sleepers (≥ 6) [Buysse et al., 1989]. Sleep duration (hours spent asleep at night), sleep efficiency (the percentage of time in bed at night spent asleep) and sleep latency (minutes taken to fall asleep at night) were also derived from answers to individual questions within the PSQI.

Sleep quality was assessed using the Jenkins Sleep Scale [Jenkins et al., 1988] at four previous phases of the Whitehall II study (Phase 5 1997–99, Phase 7 2002–04, Phase 9 2007–09, Phase 11 2012–13), as well as at the time of the MRI scan. The Jenkins Sleep Scale is a four‐item self‐report scale assessing the frequency of sleep difficulties over the preceding month. Participants were classified as having poor sleep, if they answered ‘15–20 days' or ‘21–31 days’ to any item [Lallukka et al., 2011]. The number of times participants were classified as poor sleepers according to the Jenkins Sleep Scale over all five time‐points was calculated to represent the persistence of poor sleep quality, and average score across all five time‐points was also derived.

### Health, Lifestyle, and Cognitive Assessments

A number of health and lifestyle measures previously linked to sleep quality were assessed at the same time‐point as the MRI scan. Body mass index (BMI) was calculated as weight (kg)/height^2^ (m^2^). Blood pressure was measured twice on the right arm in a sitting position (OMRON HEM‐907; OMRON Healthcare UK Ltd., Milton Keynes) with the average value used to calculate mean arterial pressure (MAP; (systolic blood pressure + 2 * diastolic blood pressure)/3)). Depressive symptoms were assessed using the Centre for Epidemiologic Studies Depression Scale (CES‐D) [Radloff, 1977]. To avoid circularity, item 11 “My sleep was restless” was excluded when calculating the CES‐D score. Medication was classified as psychotropic if listed in electronic British National Formulary legacy (BNF Legacy) Chapters 4.1 (hypnotics and anxiolytics), 4.2 (drugs used in psychoses and related disorders) or 4.3 (antidepressant drugs) [Joint‐Formulary‐Committee, 2015]. Physical activity was measured using the Community Healthy Activities Model Program for Seniors (CHAMPS) questionnaire [Stewart et al., 2001]. Metabolic Equivalent of Task (MET) minutes for moderate‐to‐vigorous physical activity were calculated from items with MET ≥ 3.0.

Participants completed a battery of cognitive tests at the same time‐point as the MRI scan, as previously outlined [Filippini et al., 2014]. For the purpose of this study, cognitive tests were divided into general cognition, assessed with the Montreal Cognitive Assessment (MoCA) [Nasreddine et al., 2005] and three domains: executive function, memory, and processing speed. The executive function domain included digit span: forward, backward and sequence [Wechsler, 2008], fluency: letter and category, and trail‐making test (TMT): B [Reitan, 1958]. The memory domain included Hopkins Verbal Learning Test Revised (HVLT‐R): total recall, delayed recall and recognition [Brandt, 1991], and Rey‐Osterrieth complex figure (RCF): immediate recall, delayed recall and recognition [Osterrieth, 1944]. The processing speed domain included TMT: A [Reitan, 1958], digit coding [Wechsler, 2008], and Cambridge Neuropsychological Test Automated Battery Reaction Time touchscreen task (CANTAB RTI; CANTABeclipse 5.0; Cambridge Cognition Ltd) simple reaction time, choice reaction time, simple movement time, choice movement time [Sahakian et al., 1993]. Where necessary, signs were reversed to ensure that higher scores represented a better performance for all tests (e.g. TMT, CANTAB reaction time).

### MRI Acquisition and Analysis

MRI data were acquired at the Oxford Centre for Functional MRI of the Brain (FMRIB) using a 3‐Tesla, Siemens Magnetom Verio (Erlangen, Germany) scanner with 32‐channel head coil. DTI scans were acquired with an echo planar imaging sequence (60 diffusion weighted directions, *b*‐value 1500s/mm^2^; five non‐diffusion weighted images, *b*‐value 0s/mm^2^, with one b0 volume acquired in the reversed phase encoded direction) with repetition time 8900ms, echo time 91.2 ms, field of view 192 mm, and voxel dimensions 2.0 mm isotropic. The susceptibility induced off‐resonance field was estimated from a pair of b0 scans using the FSL tool topup [Andersson et al., 2003]. This information was fed into the FSL tool eddy, where data was corrected for subject movement and eddy current‐induced distortions [Andersson and Sotiropoulos, 2016] and for movement induced signal voids (outliers) [Andersson et al., 2016]. Slices were classified as outliers and replaced if the signal was found to be more than three standard deviations from the Gaussian process predicted slice. If over 10 slices were identified as outliers within a volume, the volume was removed. If more than five volumes were removed, then the scan was excluded from analyses. DTIFit, part of FMRIB's Diffusion Toolbox, was used to fit a diffusion tensor model to the raw diffusion data, obtaining maps of FA, AD and RD.

Fluid attenuated inversion recovery (FLAIR) images were acquired to characterize WMHs (repetition time 9000ms, echo time 73ms, field of view 220mm, and voxel dimensions 0.9 × 0.9 × 3.0 mm^3^). WMHs were automatically segmented on FLAIR images with BIANCA (Brain Intensity AbNormality Classification Algorithm), a fully‐automated, supervised method for WMH detection, based on the k‐nearest neighbor algorithm [Griffanti et al., 2016]. BIANCA classifies the image's voxels based on their intensity and spatial features, where the intensity features were extracted from FLAIR, T1 and FA images (additional options used: local average intensity within a 3D kernel of size = 3 voxels; MNI coordinates as spatial features, with a weighting factor of 2; 24 manually segmented images as training dataset). The output image represents the probability per voxel of being WMH. The total WMH volume was calculated from the voxels exceeding a probability of 0.9 of being WMH and located within a white matter mask, adjusted for the total intracranial volume.

All image analysis was performed using tools from the FMRIB Software Library (FSL, version 5.0; http://www.fmrib.ox.ac.uk/fsl). Voxel‐wise analysis of DTI data was carried out using Tract Based Spatial Statistics (TBSS) [Smith et al., 2006], part of FSL [Smith et al., 2004]. TBSS projects all participants' FA, axial diffusivity (AD) and RD data onto a mean FA tract skeleton. The threshold for the mean FA skeleton was set at 0.2, resulting in a mask of 128,660 voxels. Global measures of mean FA, AD and RD within the skeleton mask were calculated by averaging these values across the entire white matter skeleton, and voxel‐wise statistics were also performed.

### Statistical Analysis

We employed permutation‐based methods for non‐parametric testing for all analyses [Winkler et al., 2014].

In descriptive analyses, we used the FSL tool Permutation Analysis of Linear Models (PALM) to characterise differences between current good and poor sleep quality groups, as assessed with the PSQI, in demographics, health and lifestyle measures, and cognitive measures. To reduce multiple comparisons across the eighteen cognitive outcomes, Fisher non‐parametric combination (NPC) testing was used to assess the overall p values for each cognitive domain [Winkler et al., 2016], with *P* values for individual tests reported for descriptive purposes.

To explore our primary hypotheses of differences in white matter microstructure between current good and poor sleep quality groups, we used PALM to examine group differences in global measures of WMH, FA, AD and RD, and the FSL tool Randomise examine group differences in FA, AD and RD at a voxel‐wise level. For voxel‐wise analyses, 5000 permutations were used, and the significance level set at *P* < 0.05 using threshold‐free cluster enhancement option and family‐wise error rate correction for multiple comparisons across voxels.

Next, to explore different aspects of sleep, we performed correlations between white matter measures and overall sleep quality (total PSQI score), sleep duration, sleep efficiency, and sleep latency.

Finally, we examined correlations between white matter measures and the number of times participants had poor sleep quality according to the Jenkins Sleep Scale score, measured over five time‐points, as well as correlations between white matter measures and average Jenkins Sleep Scale Score. Again, using PALM to examine global white matter measures, and Randomise for voxel‐wise analyses.

Age, sex and education were included as covariates in all analyses, except those of demographic data. Significant DTI findings were repeated with MoCA, BMI, blood pressure, depressive symptoms, current psychotropic medication, and physical activity included as additional covariates.

## RESULTS

Of 534 participants recruited for the Whitehall II Imaging Sub‐Study (2012–2014), 448 were included in analyses of current sleep quality, and 398 in analyses examining the persistence of sleep quality. Attrition of participants is provided in Supporting Information Figure e‐1. Participants excluded because of missing data were not different in age, sex, education or MoCA score, compared with participants with full datasets (Supporting Information Table e‐I).

One hundred and forty‐seven participants (33%) had PSQI scores ≥ 6 at the time of the MRI assessment and were classified as reporting poor sleep quality (Table 1, Supporting Information Fig. e‐2). Poor and good sleep quality groups were not significantly different in age or education, but the poor sleep quality group contained a greater proportion of female participants. After co‐varying for age, sex and education, the poor sleep quality group was found to display higher depressive symptoms compared with the good sleep quality group. No significant group differences were detected for BMI, blood pressure, current psychotropic medication or physical activity levels. No significant differences between poor sleep quality and good sleep quality groups were detected for MoCA, or the domains of executive function (Fisher NPC testing *P* = 0.237), memory (*P* = 0.513) or processing speed (*P* = 0.123).

**Table 1:** Group differences between current good and poor sleep quality groups

	PSQI < 6	PSQI ≥ 6	Cohen's d	*P*
Demographics				
*N* (%)	301 (67%)	147 (33%)		
Age (years)	69.0 ± 5.0	69.4 ± 5.5	0.07	0.255
Sex (*N* females, %)	49 (16%)	41 (28%)		**0.004**
Education level	3.5 ± 1.0	3.4 ± 1.1	−0.12	0.123
Health and Lifestyle				
BMI	25.9 ± 4.1	26.6 ± 4.2	0.16	0.056
Blood pressure (MAP)	96.9 ± 11.5	98.1 ± 11.2	0.13	0.101
Depressive symptoms (CES‐D, excluding sleep item)	3.3 ± 4.8	6.4 ± 6.7	**0.57**	**<0.001**
Psychotropic medication (*N*)	8 (3%)	8 (5%)		0.107
Physical activity (MET.Minutes)	1621.0 ± 1386.2	1596.8 ± 1663.6	−0.02	0.578
General cognition				
MoCA	27.3 ± 2.2	27.1 ± 2.4	0.11	0.141
Executive function				
Digit span forward	11.2 ± 2.2	10.9 ± 2.3	−0.09	0.191
Digit span backward	9.9 ± 2.6	9.8 ± 2.6	<0.01	0.495
Digit span sequence	10.3 ± 2.4	10.1 ± 2.6	−0.03	0.400
Fluency: Category	22.7 ± 5.5	21.7 ± 5.6	−0.15	0.070
Fluency: Letter	15.9 ± 4.6	15.6 ± 4.4	−0.04	0.344
Trail Making Test: B^a^	−65.0 ± 32.6	−68.4 ± 39.2	−0.04	0.348
Memory				
HVLT‐R: Total recall	28.0 ± 4.3	28.0 ± 5.0	0.01	0.461
HVLT‐R: Delayed recall	9.4 ± 2.5	9.5 ± 2.7	0.02	0.405
HVLT‐R: Recognition	10.8 ± 1.4	10.8 ± 1.4	−0.03	0.369
RCF: Immediate recall	15.9 ± 6.5	15.4 ± 6.7	−0.02	0.405
RCF: Delayed recall	15.6 ± 6.1	15.1 ± 6.3	−0.02	0.422
RCF: Recognition	8.5 ± 1.9	8.6 ± 2.0	0.10	0.169
Processing Speed				
Trail making test: A^a^	−29.6 ± 11.2	−30.3 ± 9.1	−0.02	0.432
Digit coding	63.4 ± 13.2	61.8 ± 12.8	−0.11	0.141
Simple: Reaction time^a^	−292.5 ± 63.8	−302.6 ± 68.3	−0.11	0.138
Choice: Reaction time^a^	−327.8 ± 43.8	−337.4 ± 55.5	−0.17	0.051
Simple: Movement time^a^	−264.3 ± 87.0	−275.3 ± 83.1	−0.06	0.262
Choice: Movement time^a^	−283.0 ± 74.9	−290.1 ± 81.9	−0.04	0.345
Global white matter				
WMH (%)^b^	0.405 ± 0.267	0.426 ± 0.309	0.01	0.442
FA	0.478 ± 0.017	0.474 ± 0.019	**‐0.18**	**0.037**
AD (x10^3^)	1.070 ± 0.023	1.076 ± 0.023	**0.18**	**0.041**
RD (x10^3^)	0.482 ± 0.025	0.489 ± 0.028	**0.19**	**0.034**

a Reverse scored so that higher scores indicate better performance.

b N = 443.

Values are mean ± standard deviation.

Age, sex, and education were included as covariates in all analyses, except those of demographics. Education was scored on a five‐point scale: (1) no qualifications, (2) O‐levels or equivalent, (3) A‐levels, college certificate or professional qualification, (4) degree, (5) higher degree.

**Figure 1:**
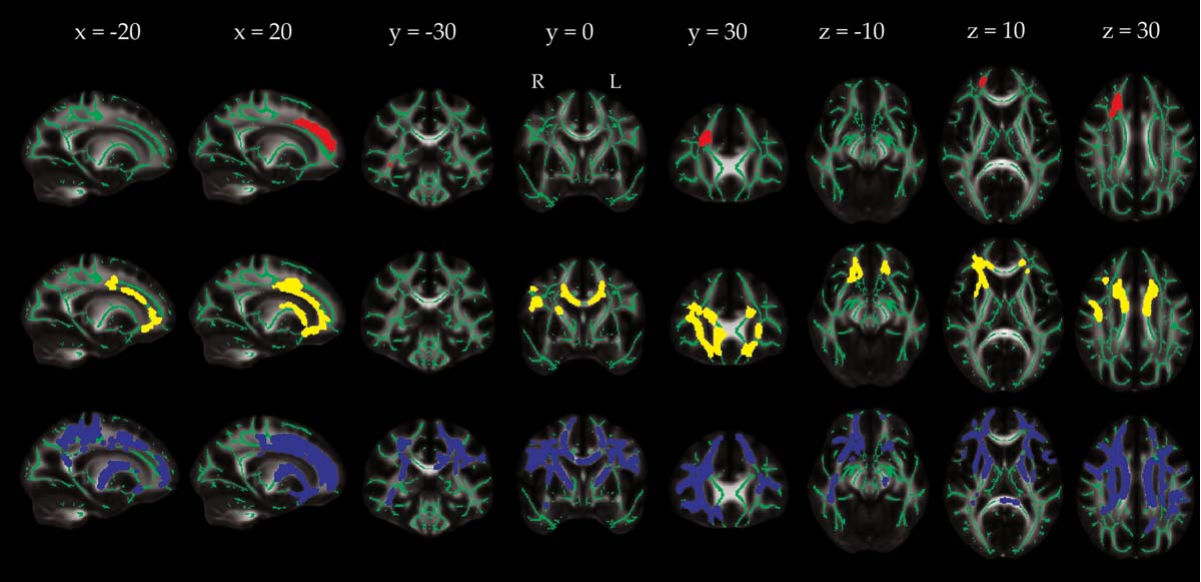
Localization of group differences in DTI measures between current good and poor sleep quality groups. Voxels displaying a significant reduction in FA (red), increase in AD (yellow) or increase in RD (blue) in the poor sleep quality group, dilated for illustrative purposes using tbss_fill, are overlaid on a green skeleton. Age, sex and education were included as covariates, with significance threshold set at *P* < 0.05, corrected for multiple comparisons across voxels. [Color figure can be viewed at http://wileyonlinelibrary.com]

After co‐varying for age, sex and education, analyses of global white matter measures showed that current poor sleep quality, as assessed by the PSQI, was associated with lower global FA, and higher global AD and RD values, with a small effect size, but not with WMH volume (Table 1). In voxel‐wise analyses, FA was significantly lower in poor sleepers in 627 voxels (0.5% of skeleton voxels), AD was significantly higher in poor sleepers in 4 895 voxels (3.8%), and RD was significantly higher in poor sleepers in 21 586 voxels (16.8%), after correction for multiple comparisons across voxels. Significant voxels predominantly fell within the frontal lobe for all measures (Fig. 1). For FA, results were primarily located within the forceps minor. For AD, significant regions extended to the anterior and superior corona radiata (bilateral), genu and body of the corpus callosum, anterior internal capsule (right), and external capsule (right), and superior longitudinal fasciculus (right). For RD, significant regions included the anterior, superior and posterior corona radiata (bilateral), genu, body and splenium of the corpus callosum, anterior and posterior internal capsule (bilateral), external capsule (bilateral), and superior longitudinal fasciculus (bilateral). After MoCA, BMI, blood pressure, depressive symptoms, psychotropic medication use, and physical activity levels were included as additional covariates, analyses of global DTI metrics averaged across the entire white matter skeleton were reduced to non‐significant levels (FA Cohen's *d* = −0.16, *P* = 0.058; AD Cohen's *d* = 0.15, *P* = 0.075; RD Cohen's *d* = 0.16, *P* = 0.062). Voxel‐wise results missed significance for FA (minimum p = 0.052), but remained significant for 13 voxels for AD (0.1% of skeleton voxels), and 6 761 voxels for RD (5.3%). Significant voxels for RD primarily fell within the anterior, superior and posterior corona radiata (right), anterior and posterior internal capsule (right), and the superior longitudinal fasciculus (bilateral) (Supporting Information Fig. e‐3).

To assess relationships with individual sleep metrics, we examined associations with overall sleep quality (total PSQI score), sleep efficiency, sleep latency and sleep duration (Supporting Information Table e‐II). Global measures of WMH volume, FA, AD, and RD were not associated with total PSQI score, sleep duration, or sleep efficiency. Sleep latency was negatively associated with global FA and positively associated with global AD and RD. In voxel‐wise analyses, total PSQI score was negatively associated with FA in 827 voxels (1% of skeleton voxels), but missed significance for AD (minimum *P* = 0.065) and RD (minimum *P* = 0.052) (Supporting Information Fig. e‐4). Sleep latency was negatively associated with FA in 6,806 voxels (5%), and positively associated with AD in 8 289 voxels (6%) and RD in 32 560 voxels (25%) (Supporting Information Fig. e‐5). Neither sleep duration nor sleep efficiency was associated with DTI metrics in voxel‐wise analyses.

To assess relationships with persistent poor sleep, the number of times participants were classified as reporting poor sleep quality on the Jenkins Sleep Scale over five time‐points over the previous 16 years was examined. 163 participants (41%) reported good sleep quality at all five time‐points, 77 (19%) reported poor sleep quality at a single time‐point, 48 (12%) reported poor sleep quality at two time‐points, 44 (11%) reported poor sleep quality at three time‐points, 29 (7%) reported poor sleep quality at four time‐points, and 37 (9%) reported poor sleep quality at all five time‐points. The number of times participants were classified as poor sleepers was not associated with WMH volume (*r* = −0.06, *P* = 0.092), global FA (*r* = 0.07, *P* = 0.069), AD (*r* = 0.01, *P* = 0.399) or RD (*r* = −0.04, *P* = 0.186). Voxel‐wise analyses similarly yielded no significant results. Average Jenkins Sleep Scale score over all five time‐points was 5.1 ± 3.4 (range 0 – 17.6), and was not associated with WMH volume (*r* = −0.061, *P* = 0.098), global FA (*r* = 0.02, *P* = 0.306), AD (*r* = 0.04, *P* = 0.208) or RD (*r* = −0.001, *P* = 0.490). Again, voxel‐wise analyses yielded no significant results.

## DISCUSSION

Sleep disturbances and decline in white matter microstructure are commonly observed in ageing populations, as well as in age‐related psychiatric and neurological illnesses. Hypothesized links between sleep and white matter have not been well established by imaging studies though. In a DTI study of 448 community‐dwelling older adults aged between 60 and 82 years we found that poor sleep quality was associated with reduced global FA and increased global AD and RD values. Widespread frontal‐subcortical tracts, encompassing regions previously reported as altered in insomnia, were affected. Differences in RD remained significant after adjustment for age, sex, education, general cognition, BMI, blood pressure, depressive symptoms, psychotropic medication use and physical activity levels – indicating that poor sleep quality may be independently associated with white matter microstructure. Caution, however, is warranted – effect sizes were small and the number of times participants reported poor sleep quality over five time‐points spanning a 16‐year period was not associated with white matter measures.

Significant voxel‐wise results were localized to white matter tracts within the frontal lobe, including the anterior and superior corona radiata, anterior internal capsule and genu and body of the corpus callosum. Despite the PSQI score amongst poor sleepers in our study averaging only 8.1 ± 2.4, compared with 11.2 ± 2.8 [Spiegelhalder et al., 2014] and 13.4 ± 3.2 [Li et al., 2016] in DTI studies of insomnia, there is notable overlap in the anatomy of our results and those described for insomnia. For example, Spiegelhalder et al. [2014] reported that insomnia patients showed reduced FA values within the right anterior capsule, with a trend for reduced FA values in the left anterior internal capsule, compared with controls. Li et al. [2016] reported reduced FA in insomnia within the right internal capsule, right corona radiata, right superior longitudinal fasciculus, body of corpus callosum, and right thalamus. In addition to the frontal‐subcortical focus, a further common finding is the preponderance of findings in the right hemisphere. In exploratory analyses, asymmetry in FA and RD values was found to be significantly different between current good sleep quality and poor sleep quality groups, in 85 voxels for FA (forceps minor) and 271 voxels for RD (forceps minor and anterior corona radiata) (Supporting Information Fig. e‐6).

It has been speculated that changes in white matter microstructure could underlie reduced cognitive function associated with poor sleep quality. However, we did not detect significant differences in general cognitive function, executive function, memory, or processing speed between good and poor sleep quality groups. While a meta‐analysis of case‐control studies reported that insomnia is associated with significant impairments of small to moderate magnitude in episodic memory, problem solving, manipulation and retention in working memory, no significant group differences were observed for general cognitive function, perceptual and psychomotor processes, procedural learning, verbal functions, different dimensions of attention and some aspects of executive functioning [Fortier‐Brochu et al., 2012]. Furthermore, the results of studies assessing the relationship between sleep quality and cognition in community‐based samples of older adults have been highly variable, with two recent reviews highlighting the inconsistency of the literature to date [Brewster et al., 2015; Yaffe et al., 2014].

PSQI reflects a range of sleep quality variables and, in exploratory analyses, we found that DTI measures were associated with total PSQI score and sleep latency, but not with sleep duration or sleep efficiency. With regard to overall sleep quality, in analyses that included only participants with insomnia, total PSQI score has previously been associated with FA within the body of the corpus callosum [Li et al., 2016]. In addition, across participants with insomnia and control participants, mean FA values within the internal capsule have been shown to be associated with Insomnia Severity Index score [Spiegelhalder et al., 2014]. With regard to sleep duration, mean FA values within the internal capsule were not associated with laboratory measured total sleep time in the same study [Spiegelhalder et al., 2014] – indicating that it may be sleep quality, rather than sleep quantity, that is most closely associated with white matter structure. Further, short sleep duration was not associated with WMH volume in older adults in the Northern Manhattan Study [Ramos et al., 2014]. However, results from the Coronary Artery Risk Development in Young Adults study indicated that short sleep is associated with an elevated ratio of WMH in the parietal lobe, and increased mean diffusivity in frontal, parietal and temporal white matter [Yaffe et al., 2016]. Sleep fragmentation has previously been associated with reduced FA and increased RD values [Baillet et al., 2017], but, to the best of our knowledge, ours is the first DTI study of community‐dwelling older adults to analyze self‐report measures of sleep latency and sleep efficiency. Given the variation in results between studies for sleep duration, further research examining individual sleep metrics is warranted before firm conclusions can be made.

In contrast to our hypotheses, we did not find that the persistence of sleep disturbances over five time‐points spanning a 16‐year period was associated with white matter measures. Note that while current sleep quality was assessed using the PSQI, historical sleep measures were assessed using the Jenkins Sleep Scale. At four‐items, the Jenkins Sleep Scale is a relatively brief assessment. Both PSQI and Jenkins Sleep Scale scores were available to assess current sleep quality, but we were unable to replicate the positive results found regarding current sleep quality when assessed using the PSQI, when using the Jenkins Sleep Scale to dichotomize participants (Supporting Information Table e‐III; *P* > 0.05 for all voxel‐wise analyses). Therefore, it is possible that the lack of significant associations with measures of persistent poor sleep may reflect limitations associated with the Jenkins Sleep Scale. In addition, since short‐term sleep deprivation has been associated with impaired performance in tests of attention, processing speed, working memory and short‐term memory [Lim and Dinges, 2010], as well as widespread fractional anisotropy decreases [Elvsåshagen et al., 2015], it is possible that our results reflect short‐term, potentially reversible, mechanisms associated with poor sleep.

As DTI scans were only acquired at a single time‐point, a causal relationship between sleep quality and white matter cannot be captured by our results. Indeed, a bi‐directional relationship (as has been proposed to exist between sleep quality and β‐amyloid [Ju et al., 2014]) may underlie our findings. In support of the hypothesis that poor sleep may be a direct cause of white matter decline, gene expression studies have shown that brain transcripts involved in myelin synthesis or maintenance are upregulated during sleep [Bellesi, 2015] and the proliferation of oligodendrocyte precursor cells has been shown to double during sleep [Bellesi et al., 2013]. While DTI metrics do not provide direct measures of white matter pathology, increased RD is consistent with myelin disruption or loss. We did not find an incremental relationship between the number of times participants were defined as poor sleepers and white matter measures though, and poor sleep may instead represent a direct consequence of white matter decline. Fronto‐subcortical tracts connect several regions that govern sleep and wakefulness [Brown et al., 2012] and disruption of such circuits may contribute to the development of sleep disturbances. Finally, it is possible that the relationship between sleep and white matter may be mediated through third factors. Importantly, we found that significant group differences in RD persisted after co‐varying for age, sex, education, MoCA, BMI, blood pressure, depressive symptoms, psychotropic medication use and physical activity levels. When considered together with the complementary results reported in DTI studies of insomnia that included strict exclusion criteria regarding psychiatric co‐morbidity, our results indicate that while third factors may well contribute to observed relationships between sleep and white matter, an independent pathway is also likely.

In evaluating the potential of modifiable factors that could help maintain, or even improve, white matter microstructure in ageing, it is important to consider not only the likely directionality of the relationship, but also the prevalence of the modifiable factor in question, and the accompanying effect size. For example, in our current work, approximately one in three participants met criteria for current poor sleep quality, but effect size for global DTI metrics was small, at ∼0.2. It should be borne in mind, though, that this effect size relates to global DTI metrics, averaged across the entire white matter skeleton. As our voxel‐wise analyses show, effects are strongest in specific white matter tracts and so the global effect size reported here will likely underestimate the strength of associations within fronto‐subcortical pathways. Nevertheless, in comparison, we found that sub‐threshold depressive symptoms were associated with effect sizes of 0.4–0.5 in an overlapping sample, but only affected one in ten participants [Allan et al., 2016]. While interventions targeting a single modifiable factor would undoubtedly be informative with regard to understanding the biological mechanisms that underlie observational results, multidomain interventions may hold the greatest promise for future trials aimed at improving white matter microstructure.

Our study has several strengths, including a relatively large sample size compared to previous DTI studies examining insomnia, a battery of established cognitive tests spanning three major cognitive domains, and longitudinal data on sleep quality over 5 time‐points spanning, on average, 16 years. However, there are also a number of limitations. For example, all sleep measures examined were self‐reported. Discrepancies between subjective and objective sleep measures have been described [Buysse et al., 2008], and objective measures of sleep may be more sensitive to cognitive deficits [Cavuoto et al., 2016]. Also, the high proportion of men and high education level within our sample will limit the generalizability of results.

In conclusion, we found that current poor sleep quality was associated with a pattern of reduced FA and increased AD and RD in a sample of community‐dwelling older adults. Differences in RD survived after adjustment for demographics, general cognition, health, and lifestyle measures; indicating that poor sleep quality may be independently associated with white matter microstructure. However, small effect sizes may limit the promise of poor sleep quality as a stand‐alone modifiable factor that could help maintain, or even improve, white matter microstructure in ageing.

## Supporting information

Supporting Information
