# Supplementary material for: Associations between self‐reported sleep quality and white matter in community‐dwelling older adults: A prospective cohort study
Source: Hum Brain Mapp. 2017 Jul 26;38(11):5465–73. doi: 10.1002/hbm.23739 (PMC5655937; doi:10.1002/hbm.23739)
Supplement: Supplementary file 1 — Supporting Information [file HBM-38-5465-s001.docx]

**Figure e-1. Attrition of participants**

479 Participants eligible

55 Excluded

48 Neurological exclusion

7 Sleep exclusion

448 Participants included in

current sleep quality analyses

31 Missing data

50 Missing data

398 Participants included in

cumulative sleep quality analyses

534 Participants randomly recruited for

Whitehall II Imaging Sub-Study (05/12 – 12/14)

**Table e-I. Comparison of participants included and those excluded due to missing data**

^a^ corrected for multiple contrasts

|  | **Complete Data** | **Missing Data** | **Cohen’s d** | **p^a^** |
| --- | --- | --- | --- | --- |
| N | 398 | 81 |  |  |
| Age (years) | 69.4 ± 5.2 | 68.8 ± 5.1 | -0.11 | 0.316 |
| Sex (N females, %) | 79 (20%) | 18 (22%) | 0.06 | 0.547 |
| Education | 3.5 ± 1.1 | 3.3 ± 1.2 | -0.16 | 0.188 |
| MoCA | 27.3 ± 2.3 | 27.0 ± 2.3 | -0.12 | 0.316 |

**Figure e-2. Distribution of PSQI Scores.**

**Figure e-3. Localisation of associations between total PSQI and FA.**

Voxels displaying a significant negative association between total PSQI and FA, displayed in red and dilated for illustrative purposes using tbss_fill, are overlaid on a green skeleton. Age, sex and education were included as covariates, with significance threshold set at p < 0.05, corrected for multiple comparisons across voxels.

**Table e-II. Associations with individual sleep metrics.**

Values are mean ± standard deviation [range].

|  |  | WMH | | FA | | AD | | RD | |
| --- | --- | --- | --- | --- | --- | --- | --- | --- | --- |
|  |  | Correlation (r) | p-value | Correlation (r) | p-value | Correlation (r) | p-value | Correlation (r) | p-value |
|  |  |  |  |  |  |  |  |  |  |
| Total PSQI score | 4.7 ± 3.0 [0 – 17] | -0.02 | 0.371 | -0.06 | 0.093 | 0.06 | 0.081 | 0.06 | 0.090 |
| Sleep duration (hours) | 7.0 ± 1.0 [3.5 – 9] | 0.05 | 0.158 | 0.02 | 0.331 | -0.01 | 0.410 | -0.01 | 0.445 |
| Sleep efficiency (%) | 83.7 ± 11.6 [34 – 100] | 0.03 | 0.226 | 0.02 | 0.299 | -0.01 | 0.402 | -0.01 | 0.384 |
| Sleep latency (minutes) | 18.9 ± 18.8 [0 – 50] | 0.01 | 0.415 | -0.09 | **0.021** | 0.08 | **0.034** | 0.09 | **0.021** |

**Figure e-4. Localisation of associations between sleep latency and DTI measures.**

Voxels displaying a significant negative association between sleep latency and FA (red), or a significant positive association between sleep latency and AD (yellow) or RD (blue), dilated for illustrative purposes using tbss_fill, are overlaid on a green skeleton. Age, sex and education were included as covariates, with significance threshold set at p < 0.05, corrected for multiple comparisons across voxels.

**Figure e-5. Localisation of group differences in DTI measures between current poor and good sleep quality groups, following addition of covariates examining general cognition, health and lifestyle measures.**

Voxels displaying a significant increase in AD (yellow) or RD (blue) in the poor sleep quality group, dilated for illustrative purposes, are overlaid on a green skeleton. Age, sex, education, MoCA, BMI, blood pressure, depressive symptoms, psychotropic medication use, and physical activity levels were included as covariates, with significance threshold set at p < 0.05, corrected for multiple comparisons across voxels.


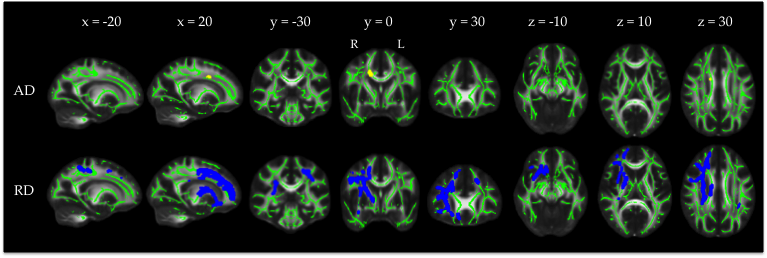


**Figure e-6. Localisation of group differences in asymmetry in DTI measures between current poor and good sleep quality groups.**

In order to test for differences in asymmetries in DTI metrics between current good sleep quality and current poor sleep quality groups, tbss_sym was used to generate left-minus-right images for FA, AD and RD. The significance threshold was set at p < 0.05, corrected for multiple comparisons across voxels. Voxels displaying a significant difference between groups in left-minus-right for FA (red) or RD (blue), dilated for illustrative purposes using tbss_fill, are overlaid on a green skeleton. Mean left-minus-right values within significant voxels are also displayed. No significant differences between groups were detected for AD.


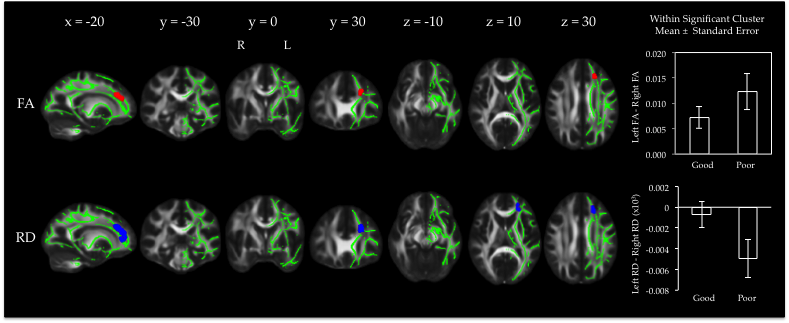


**Table e-III. Group differences in white matter measures between current good and poor sleep quality groups, defined using the Jenkins Sleep Scale.**

Values are mean ± standard deviation. ^a^ N = 443

|  | **Good Sleep** | **Poor Sleep** | **Cohen’s d** | **p** |
| --- | --- | --- | --- | --- |
| N | 309 (69%) | 139 (31%) |  |  |
|  |  |  |  |  |
| *White Matter* |  |  |  |  |
| WMH (%) ^a^ | 0.404 ± 0.271 | 0.430 ± 0.302 | 0.01 | 0.481 |
| FA | 0.477 ± 0.017 | 0.477 ± 0.018 | -0.15 | 0.076 |
| AD (x10^3^) | 1.072 ± 0.023 | 1.074 ± 0.024 | 0.01 | 0.473 |
| RD (x10^3^) | 0.484 ± 0.026 | 0.485 ± 0.027 | 0.10 | 0.168 |
